# MiR-499 inhibited hypoxia/reoxygenation induced cardiomyocytes injury by targeting SOX6

**DOI:** 10.1007/s10529-019-02685-3

**Published:** 2019-05-10

**Authors:** Yujie Shi, Yunfeng Han, Lili Niu, Junxia Li, Yundai Chen

**Affiliations:** 1 Department of Cardiology, Chinese PLA General Hospital, No. 28 Fuxing Road, Beijing, 100853 China; 2 Cardiovascular Disease Institute, PLA Army General Hospital, Beijing, China

**Keywords:** Cardiomyocytes, miR-499, Hypoxia/reoxygenation, SOX6

## Abstract

**Objective:**

MiR-499 has been reported to be expressed only in cardiomyocytes, and its expression would increase after acute myocardial infarction (AMI). miR-499 plays a role in the process of cardiomyocytes injury induced by hypoxia/reoxygenation (H/R), however, it still remains unclear.

**Results:**

Hypoxia inhibited miR-499-5p expression and H/R induced apoptosis. SOX6 was a target gene of miR-499-5p, and high expression of miR-499-5p inhibited the expression of SOX6. MiR-499-5p reduced H9c2 cells injury by inhibiting the expression of SOX6, overexpression of which could reverse the effect of miR-499-5p on H9c2 cells. MiR-499-5p inhibited the levels of LDH and MDA, while overexpression of miR-499-5p inhibited H/R-induced cell apoptosis. MiR-499-5p could up-regulate the level of Bcl-2 and down-regulate the expression levels of Bax and caspase-3. However, SOX6 partially reversed these effects of miR-499-5p.

**Conclusion:**

We proved that miR-499-5p inhibited H/R-induced cardiomyocytes injury by targeting SOX6. Our results suggested that miR-499-5p/SOX6 pathway may present a potential therapeutic target for the treatment of AMI.

## Introduction

As a type of myocardial necrosis caused by acute continuous ischemia and hypoxia in coronary arteries, acute myocardial infarction (AMI) is characterized by an acute onset and a high mortality rate (Du et al. 2015). Percutaneous coronary interention (PCI) and coronary artery bypass graft surgery (CABG) can recover blood supply in the myocardium. However, the injury of ischemic myocardial tissue will be aggravated in case that blood supply in myocardium is recovered, therefore causing myocardial ischemia reperfusion injury (Hernandez-Resendiz et al. 2018). Myocardial cell injury could be caused by cell necrosis and apoptosis (Feng et al. 2016). Hypoxia can damage the microenvironment homeostasis, leading to myocardial cell apoptosis and aggravating the condition of AMI (Zhang et al. 2017). Therefore, an effective prevention of myocardial cell injury caused by hypoxia is the key to the treatment of AMI, and understanding the molecular mechanisms of AMI is critical to the development of new therapies.

MicroRNAs (miRNAs) are a group of non-coding, single-stranded RNA molecules with a length of 18–25 nucleotides encoded by endogenous genes, and miRNAs are widely found in eukaryotes (Chen et al. 2017). MiRNAs can regulate gene expression by binding to the 3′-untranslated region (3′UTR) of the target gene messenger RNA (mRNA) and downregulating mRNA translation or by promoting mRNA degradation at the post-transcriptional level (Zhu et al. 2016). Studies have shown that miRNAs played crucial roles in the pathogenesis of a variety of cardiovascular diseases (Greco et al. 2014; Liang et al. 2018). Apoptosis of cardiomyocytes is an important cellular event in AMI (Wang et al. 2016a), and several studies found that some miRNAs had protective effects on the myocardial injury induced by hypoxia/reoxygenation (H/R) (Huang et al. 2017; Ren et al. 2018; Wu et al. 2015). However, the mechanism of such a protective function has not been fully elucidated.

Previous studies have confirmed that the expressions of miR-1/-133a/-499 in the serum of AMI patients were increased significantly (Wang et al. 2010). MiR-499 was found to be expressed only in cardiomyocytes. Moreover, an increase of miR-499 expression was observed in AMI patients, whereas a reduction of this expression was found in normal hearts. (Agiannitopoulos et al. 2018; Zhang et al. 2015). Xin et al. found that miR-499 could be used as a potential predictive biomarker for AMI (Xin et al. 2016). MiR-499-5p, which is an evolutionarily and highly conserved specific microRNA, is highly expressed in ventricles. In addition, Wang et al. demonstrated that miR-499-5p could protect H_2_O_2_-induced cardiomyocytes (Wang et al. 2014a). However, the role of miR-499-5p expression level in the process of myocardial cells H/R injury remains unclear. Lactate dehydrogenase (LDH) and malondialdehyde (MDA) are common markers of myocardial injury. LDH is a catalytic enzyme involved in the conversion of pyruvate and lactic acid with cytoplasm, and by detecting the leakage rate of cell culture medium LDH, the degree of cell injury can be therefore measured. MDA content can reflect the severity of cell lipid membrane damage caused by oxygen free radical attack.

SOX gene family is a type of transcription factor coding gene with a highly conserved HMG-box sequence. SOX gene family can be divided into 10 subgroups (A–J), and SOX6 is an important member of the SOX D subgroup (Bowles et al. 2000). Previous studies have found that miR-499 could inhibit the apoptosis of P19CL6 cells by the regulation of SOX6 at the late stage of cardiac differentiation (Li et al. 2013). It has also been found that miR-499 protected cardiomyocytes against LPS-induced apoptosis by targeting SOX6 and programmed cell death protein 4 (PDCD4) (Jia et al. 2016). However, whether miR-499 protected cardiomyocytes against H/R-induced myocardial injury by regulating SOX6 remains unclear. Thus, in this study, we explored the role and mechanism of miR-499 in the myocardial cell injury induced by H/R.

## Materials and methods

### H9c2 cells culture

Rat H9c2 cells were obtained from the Cell Bank of Chinese Academy of Sciences (Shanghai, China). Dulbecco’s modified Eagle’s medium (DMEM) and fetal bovine serum (FBS) were purchased from Invitrogen (Carlsbad, USA). H9c2 cells were cultured in DMEM medium containing 10% FBS at 37 °C in an incubator (Thermo Fisher Scientific, Rockford, USA) with 5% CO_2_.

### H/R injury model generation

According to the experimental requirements, H9c2 cells were treated with H/R. The cells were induced by hypoxia in a modular incubator (Thermo Fisher Scientific, Rockford, USA) with 1% O_2_, 94% N_2_ and 5% CO_2_ for 6 h. Next, reoxygenation was performed for 3 h in a modular incubator (Thermo Fisher Scientific, Rockford, USA) with 5% CO_2_ at 37 °C. H9c2 cells under normoxia were treated as control in the study.

### Cell groups

To explore the effect of H/R on H9c2 cells, the H9c2 cells were divided into control (normoxia) and H/R group (H/R induced), and the cells apoptosis were detected using flow cytometry. To study the effect of miR-499-5p on SOX6, the H9c2 cells were divided into 4 groups as follows: inhibitor group (transfected with miR-499-5p inhibitor), mimic group (transfected with miR-499-5p mimic), mock group (transfected with scrambled sequence) and control group. H9c2 cells were divided into 3 groups as follows: mimic + SOX6 group (transfected with mimic and pcDNA3.1-SOX6), mimic + negative control (NC) group (transfected with mimic and pcDNA3.1) and control group to help further explore the relation between miR-499-5p and SOX6. To explore the effects of miR-499-5p and SOX6 on hypoxia-induced myocardial cells, H9c2 cells were divided into 6 groups as follows: mimic + NC + hypoxic group (transfected with mimic and pcDNA3.1, hypoxic treatment for 6 h), mimic + SOX6 + hypoxic group (transfected with mimic and pcDNA3.1-SOX6, hypoxic treatment for 6 h), mimic + hypoxia group (transfected with mimic and hypoxic treatment for 6 h), mock + hypoxia group (transfected with scrambled sequence and hypoxic treatment for 6 h), hypoxia group (hypoxic treatment for 6 h) and control group.

### Transfection

H9c2 cells were digested using trypsin (Gibco, Carlsbad, USA) and counted the day before transfection. The H9c2 cells were cultured to a density of 90% in an incubator with 95% O_2_ and 5% CO_2_ at 37°C the day of transfection. The mock, miR-499-5p mimic and inhibitor were obtained from GenePharma (Shanghai, China) and listed in Table 1. MiRNAs were transfected into H9c2 cells by Lipofactamine 2000 (Invitrogen, Carlsbad, CA, USA) as previously described (Jia et al. 2016). The pcDNA3.1-SOX6 plasmid and vector were obtained from GenePharma (Shanghai, China), and empty pcDNA3.1 plasmid was used as negative control. H9c2 cells were incubated in an incubator with 5% CO_2_ at 37 °C. All transfections were performed before the formation of H/R-induced injury, and then cultured for 48 h, and the expression of miR-499-5p was determined.

**Table 1:** Sequences for cell transfection

Group name	Sequences
miR-499-5p mimic	Sense: 5′-UUAAGACUUGCAGUGAUGUUU-3′
	Antisense: 5′-AAACAUCACUGCAAGUCUUAA-3′
miR-499-5p inhibitor	5′-AAACAUCACUGCAAGUCUUAA-3′
Mock control	Sense: 5′-UCACAACCUCCUAGAAAGAGUAGA-3′
	Antisense: 5′-UCUACUCUUUCUAGGAGGUUGUGA-3′
miR-499	Forward: 5′-TTAAGACTTGCAGTGATGTTT-3′
	Reverse: 5′- GAACATGTCTGCGTATCTC-3′
SOX6	Forward: 5′-CACUUGUCAGUACCAUUCATT-3′
	Reverse: 5′-UGAAUGGUACUGACAAGUGTT-3′
GAPDH	Forward: 5′-CATCACTGCCACCCAGAAGACTG-3′
	Reverse: 5′-ATGCCAGTGAGCTTCCCGTTCAG-3′

### Quantitative real-time polymerase chain reaction (RT-qPCR)

The mRNA expressions of miR-499-5p and SOX6 were detected by RT-qPCR. TRIzol kit (Invitrogen, Carlsbad, USA) was applied to extract the total RNA, and Nanodrop 2000 (Thermo Scientific, Wilmington, USA) was applied to detect the concentration and purity of RNA. PrimeScript RT kit (TaKaRa, Dalian, China) was applied to perform the reverse transcription of the miRNAs into cDNA. Reverse transcription reaction conditions were set at 37 °C for 15 min and reverse transcriptase inactivation conditions were set at 85 °C for 15 s. RT-qPCR was performed in ABI 7500 real-time RT-PCR system with the SYBR-Green Universal qPCR Master Mix (Applied Biosys-tems, Darmstadt, Germany). The reactions were conducted as follows: 95 °C for 2 min, followed by 40 cycles of three-step (95 °C for 15 s, 60 °C for 15 s, 72 °C for 20 s), 72 °C for 7 min. The primers of miR-499, SOX6 and GAPDH were listed in Table 1. GAPDH was the internal reference, and the expression level of U6 was the internal reference. The formula 2^−ΔΔCt^ was applied to determine the mRNA expression.

### Dual luciferase reporter

The target genes of miR-499-5p were predicted by Targetscan7.2. The wild type (WT) of the SOX6 gene was amplified using PCR, and SOX6-WT was cloned into the psiCHECK™-2 vector (Promega, Madison, USA). The mutant type (MUT) of SOX6 was constructed by Quick Change Site Directed Mutagenesis kit (Stratagene, La Jolla, USA) and treated as a negative control. Dual luciferase reporter gene assay kit (Promega, Madison, USA) was performed to detect the luciferase activity on a LD400 luminometer (Promega, Madison, USA).

### Cell apoptosis analysis

Flow cytometry was used to detect cell apoptosis. The annexin V-fluorescein isothiocyanate (FITC) apoptosis detection kit (Bender Med System, CA) was used to determine apoptosis as previously described (Wu et al. 2015). H9c2 cells were first digested with 0.25% trypsin and collected by centrifugation and then washed by cold phosphate buffer saline (PBS; Invitrogen, Carlsbad, USA) and finally centrifuged at 4 °C (200×*g*, 10 min). H9c2 cells were resuspended in 200 µL binding buffer with 10 µL annexin-V-FITC and 5 µL propidium iodide and incubated in the dark for 30 min at room temperature, and 300 µL binding buffer was then added. Finally, the H9c2 cells apoptosis was detected using BD FACSCalibur flow cytometer, and data analysis was performed by the Cell Quest software (BD Biosciences). The early apoptotic cells were in the lower right quadrant, while advanced apoptotic cells were in the upper right quadrant. The percentage of apoptotic cells is the sum of the percentages of early apoptotic cells and advanced apoptotic cells.

### Enzyme-linked immuno sorbent assay (ELISA)

The concentrations of LDH and MDA in the medium after hypoxia induction were determined using LDH Assay Kit (ab65393, Abcam) and MDA Assay Kit (ab238537, Abcam), respectively. The enzyme labeling reagent and a developer were added according to the operation instructions, and the reaction was terminated by adding one stop solution. Optical densities at 450 nm were determined by a microplate reader (Model 680, Bio-Rad, USA) and the concentrations were measured.

### Western blot assays

The cells were dissolved in RIPA lysis buffer with 1% phenylmethylsulfonyl fluoride and centrifuged (12,000×*g*, 15 min) at 4 °C. Pierce™ BCA protein assay kit (Thermo Fisher Scientific, Rockford, USA) was used to detect the protein concentration, and the SDS-PAGE was used to separate the proteins. Afterwards, the proteins were transferred onto a PVDF membrane and blocked with 5% fat-free milk at room temperature for 1 h. Next, the membrane was incubated with primary antibodies (anti-SOX6, ab30455, Abcam, 1:1000; anti-GAPDH, ab8245, Abcam, 1:1000; anti-Bcl-2, ab196495, Abcam, 1:1000; anti-Bax, ab53154, Abcam, 1:1000; anti-Cleaved-caspase 3, #9661, CST, 1:1000) at 4 °C overnight and washed with TBST for three times (5 min/time) and then incubated with the horseradish peroxidase conjugated secondary antibody (goat anti-rabbit IgG; ab205718; Abcam, 1:1000) for 1 h at room temperature. Chemiluminescence detection was performed by chemiluminescence kit (Pierce Chemical, Rockford, USA).

### Statistical analysis

The data were expressed as the mean ± standard deviation. Statistical analysis was conducted applying SPSS 19 (SPSS, Inc., Chicago, USA). The variances between different groups were analyzed by *t* test or one-way analysis of variance. *p *< 0.05 was considered as a statistically significant difference.

## Results

### MiR-499-5p was down-regulated in hypoxia-induced H9c2 cells

**Fig. 1:**
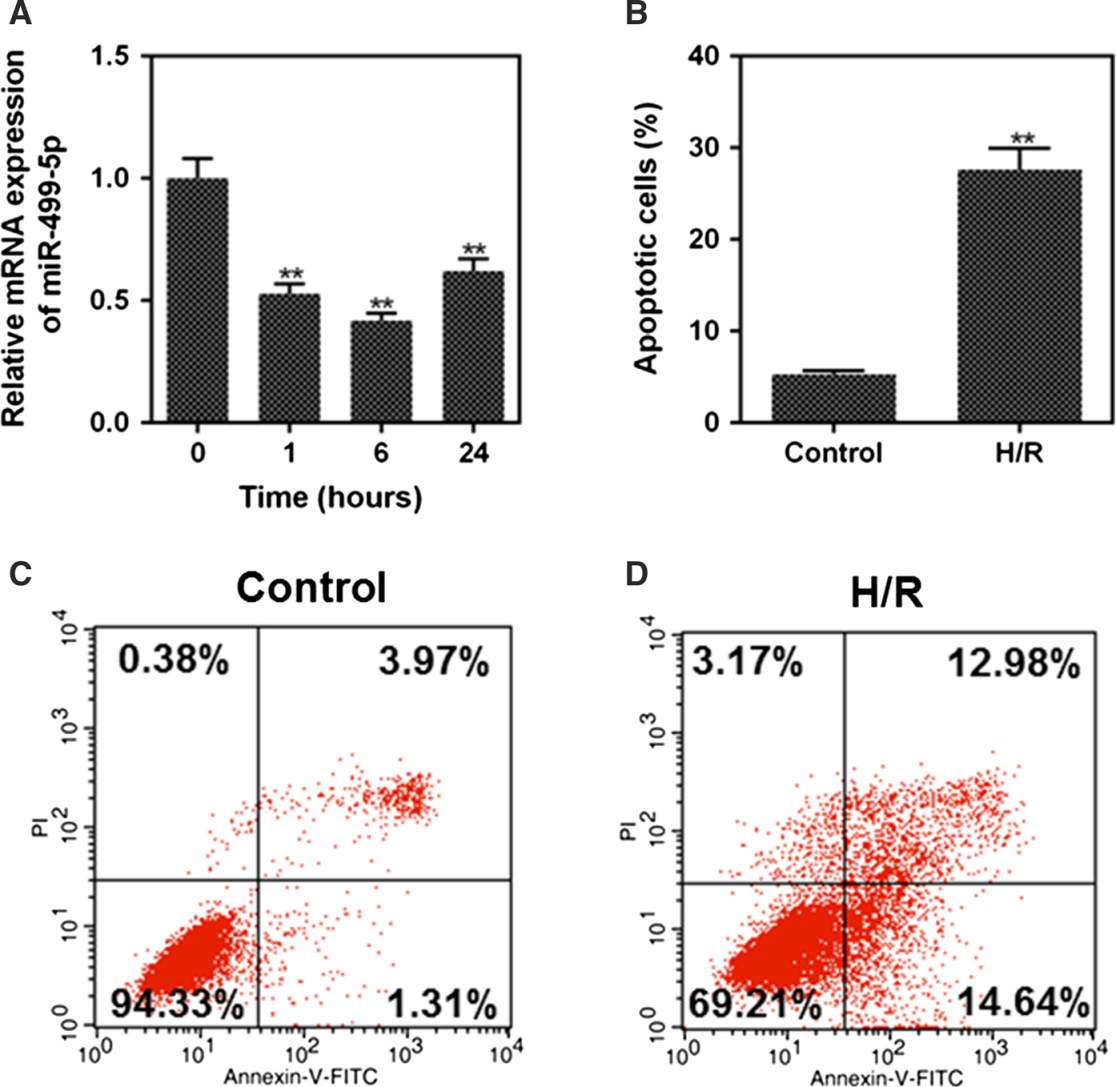
The effect of hypoxia on miR-499-5p expression and apoptosis. **a** The mRNA expression of miR-499-5p in the H9c2 cells after being induced by hypoxia for 0, 1, 6, 24 h. (^**^P < 0.01 vs. 0 h). (**b**–**d**) Cell apoptosis in the control and H/R group was detected by flow cytometry. (^**^P < 0.01 vs. control)

In the study, the hypoxia time points were selected as 0, 1, 6, 24 h as previously described (Li et al. 2016), and the mRNA expressions of miR-499-5p under different time points were determined by performing RT-qPCR. miR-499-5p expressions at 1, 6 and 24 h after the induction of hypoxia were lower than that without the induction of hypoxia (0 h), and the expression of miR-499-5p at 6 h was the lowest (Fig. 1a, *p *< 0.01). Therefore, 6 h was selected as the hypoxia treatment time in subsequent experiments.

### H/R induced H9c2 cells apoptosis

The selection of anoxia and reoxygenation time was determined referring to previous studies (Li et al. 2016; Zhang et al. 2014). Cells were induced by hypoxia for 6 h and reoxygenated for 3 h, and the apoptosis in control group and H/R group were detected by flow cytometry. The apoptosis rate of H/R group was higher than that of control group (Fig. 1b–d, *p *< 0.01).

### SOX6 was a target gene of miR-499-5p

**Fig. 2:**
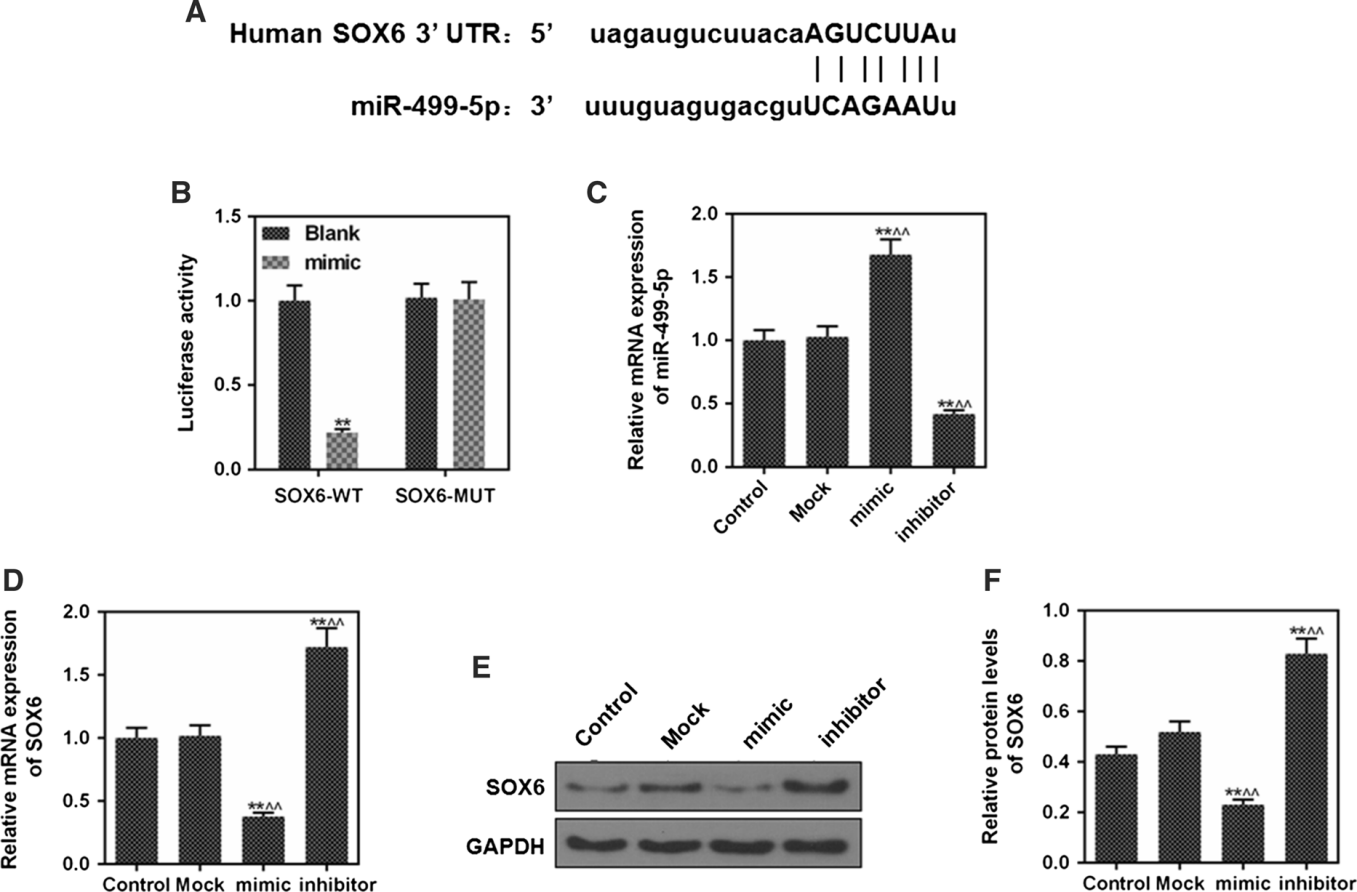
SOX6 was detected as a target of miR-499-5p. **a** The binding site of SOX6 3′-UTR with miR-499-5p. **b** The luciferase activity of reporter vector with WT or MUT SOX6 3′–UTR in cells (^**^P < 0.01 vs. blank). **c** The mRNA expressions of miR-499-5p in control, mock, mimic and inhibitor group were determined by RT-qPCR. **d** The mRNA expressions of SOX6 in control, mock, mimic and inhibitor group were detected by RT-qPCR. (E, F) The SOX6 protein levels in control, mock, mimic and inhibitor group were determined by western blot. GAPDH was as an internal reference. ^**^P < 0.01 vs. control, ^^^^*P* < 0.01 vs. mock

Targetscan7.2 suggested that SOX6 was the target gene of miR-499-5p in H2c9 cells, and that the 3′-UTR of the SOX6 mRNA contained a binding site for miR-499-5p (Fig. 2a). The miR-499-5p could decrease the luciferase activity of SOX6 3′UTR- WT reporter vector (Fig. 2b, *p *< 0.01), while miR-499-5p had no effect on luciferase activity of SOX6 3′UTR-MUT reporter vector. The results confirmed that SOX6 was the target gene of miR-499-5p. In addition, we detected the expressions of miR-499-5p in control, mock, mimic and inhibitor group, and data showed no significant difference in miR-499-5p expression between the control and the mock group, however, it was noted that the mRNA expression of miR-499-5p was increased in mimic group and was decreased in inhibitor group (Fig. 2c, *p *< 0.01), suggesting that miR-499-5p was successfully transfected into H2c9 cells.

### MiR-499-5p down-regulated SOX6 expression

**Fig. 3:**
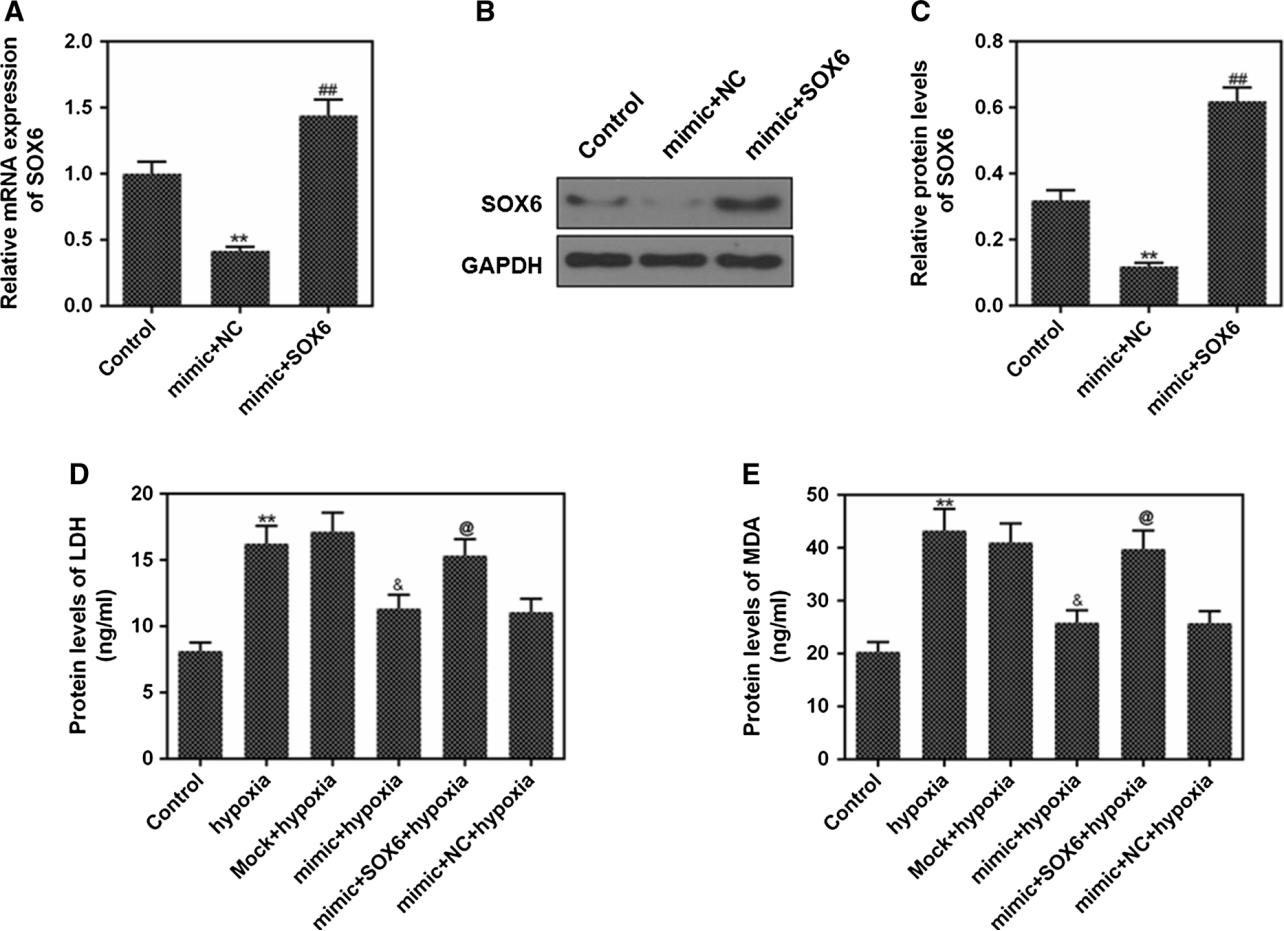
SOX6 induced myocardial cell injury. **a** RT-qPCR was performed to detect the mRNA expressions of SOX6 in control, mimic + NC, mimic + SOX6. **b**, **c** The protein levels of SOX6 in control, mimic + NC and mimic + SOX6 were determined by western blot. **d**, **e** The protein levels of LDH and MDA in control, hypoxia, mock + hypoxia, mimic + hypoxia, mimic + SOX6 + hypoxia and mimic + NC + hypoxia. GAPDH was as an internal reference. ^**^P < 0.01 vs. control, ^##^*P* < 0.01 vs. mimic + NC, ^&&^*P* < 0.01 vs. hypoxia, ^@@^*P* < 0.01 vs. mimic + hypoxia

The miR-499-5p overexpression inhibited the SOX6 mRNA and protein expressions, and low expression of miR-499-5p promoted the expression of SOX6 (Fig. 2d–f, *p *< 0.01). The mRNA and protein expressions of SOX6 in mimic + SOX6 group were increased, compared with mimic + NC group (Fig. 3a–c, *p *< *0.01*).

### SOX6 could partially reverse the inhibitory effect of miR-499-5p on LDH and MDA

To detect the effect of miR-499-5p on H9c2 cells injury, the levels of LDH and MDA in each group were determined by ELISA. LDH and MDA levels were up-regulated by hypoxia. The levels of LDH and MDA in mimic + hypoxia group were decreased, compared with those in hypoxia group (Fig. 3d, e, *p *< 0.05), indicating that miR-499-5p inhibited the LDH and MDA levels and reduced H9c2 cell injury. However, LDH and MDA levels in mimic + SOX6 + hypoxia group were increased, compared with those in mimic + hypoxia group (Fig. 3d, e, *p *< 0.05), indicating that SOX6 partially reversed the inhibitory effect of miR-499-5p on LDH and MDA.

### SOX6 was involved in myocardial cell apoptosis induced by H/R mediation

**Fig. 4:**
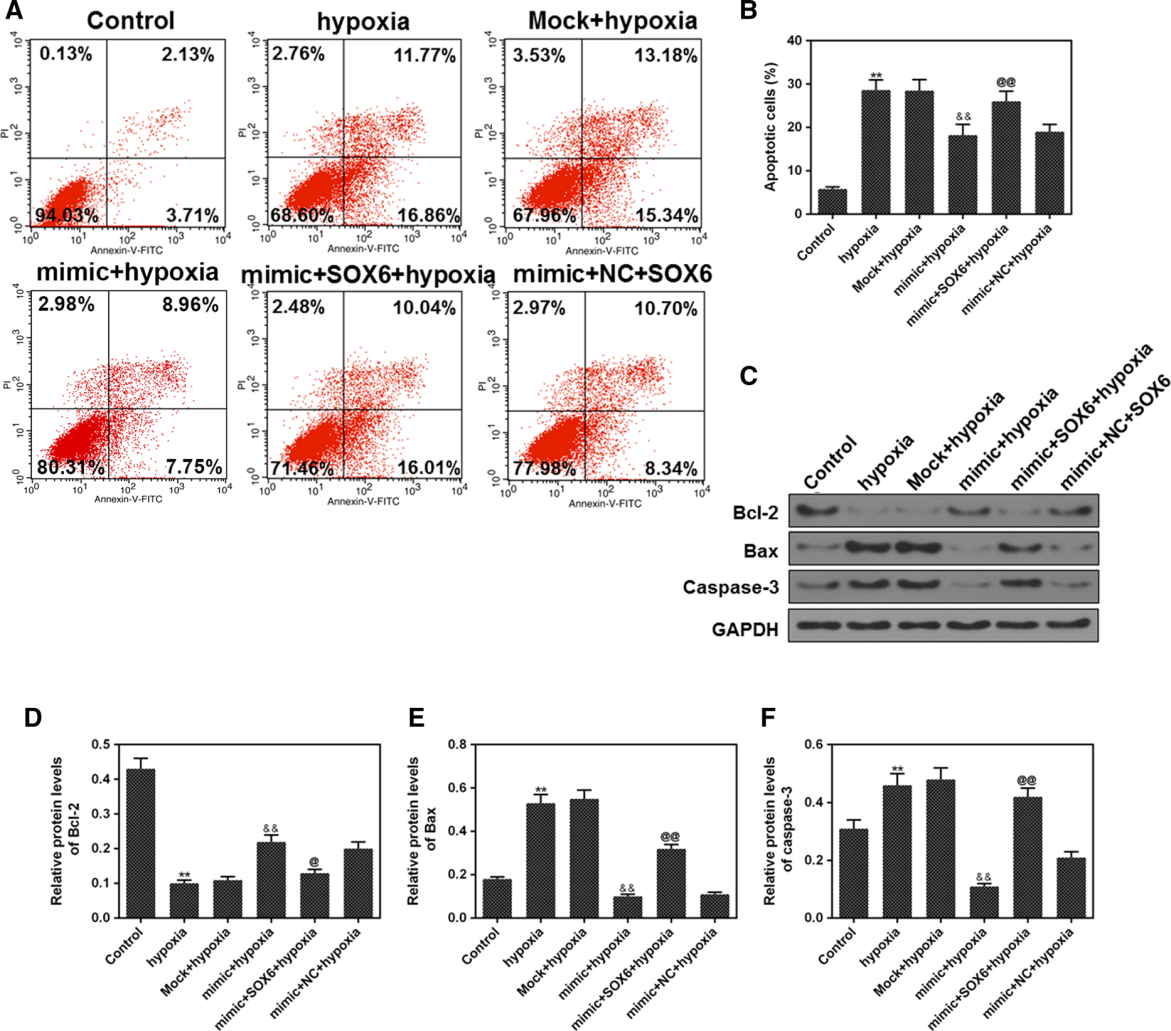
MiR-499-5p overexpression played an anti-apoptosis role. **a**, **b** Apoptosis was detected in control, hypoxia, mock + hypoxia, mimic + hypoxia, mimic + SOX6 + hypoxia and mimic + NC + hypoxia group by flow cytometry. (C-F) The levels of Bcl-2, Bax and caspase-3 were determined in control, hypoxia, mock + hypoxia, mimic + hypoxia, mimic + SOX6 + hypoxia and mimic + NC + hypoxia group by western blot. GAPDH was as an internal reference. ^**^P < 0.01 vs. control, ^&&^*P* < 0.01 vs. hypoxia, ^@@^*P* < 0.01 vs. mimic + hypoxia

After 6 h of hypoxia induction, the apoptosis rate in each group (control, hypoxia, mock + hypoxia, mimic + hypoxia, mimic + SOX6 + hypoxia, mimic + NC + hypoxia) was detected by flow cytometer. The H9c2 cells apoptosis was increased in hypoxia group, and the apoptosis rate of cells in the mimic + hypoxia group was decreased, compared with the hypoxia group, and apoptotic cells in mimic + SOX6 + hypoxia group was significantly more than those in mimic + hypoxia group (Fig. 4a, b, *p *< 0.01). Hypoxia could down-regulate the protein level of Bcl-2 and up-regulate the protein levels of Bax and caspase-3. The result showed that miR-499-5p up-regulated the protein level of Bcl-2 and down-regulated the protein levels of Bax and caspase-3. In comparison with mimic + hypoxia group, Bcl-2 level of mimic + SOX6 + hypoxia group was down-regulated, while the protein levels of caspase-3 and Bax in mimic + SOX6 + hypoxia group were up-regulated (Fig. 4c–f, all *p *< 0.01). This indicated that miR-499-5p could inhibit the H9c2 cell apoptosis rate, and SOX6 could partially reverse the inhibitory effect of miR-499-5p.

## Discussion

AMI is one of the most frequent clinical emergencies. Cardiomyocyte apoptosis, which plays an important role in AMI, occurs in myocardial tissue after ischemia/reperfusion injury. Studies have reported that miRNAs participated in the regulation of myocardial apoptosis (Liu et al. 2017; Zhang et al. 2014). In the present study, we revealed that miR-499-5p inhibited H/R-induced cardiomyocytes injury by targeting SOX6, which could partially reverse the protective effect of miR-499-5p on cardiomyocytes. These results indicated that miR-499-5p/SOX6 could be a potential therapeutic target of AMI and could promote the development of novel targeted therapies.

It was reported that changes occurred to miR-499 expression in myocardial tissue or cells under the stimulation of ischemia, hypoxia or oxidative stress (Matkovich et al. 2012; Shieh et al. 2011; Wang et al. 2014a). Cheng et al. reported that miR-449a could protect H9c2 cardiomyocyte against H/R-induced injury via targeting Notch-1 signaling pathway (Cheng et al. 2018). MiR-499-5p is a member of myosin-encoded miRNAs (Olivieri et al. 2013), and that it could be differentially regulated and functioned during the development of the heart (Sluijter et al. 2010). Here, we explored the role of miR-499-5p in H/R induced-injury in cardiomyocytes and its molecular mechanism. In our study, the result showed that hypoxia inhibited miR-499-5p expression, which was consistent with the results of Li et al. (Li et al. 2016). In addition, we found that the apoptosis induced by H/R was remarkably increased, suggesting that H/R induced cardiomyocytes apoptosis.

MiRNA could regulate multiple target genes to exert their biological functions. Previous studies have found that miR-499-5p attenuated mitochondrial fission and cell apoptosis by regulating p21 in doxorubicin cardiotoxicity (Wan et al. 2018). In addition, it has been reported that the target genes of miR-499-5p in the anti-apoptosis process of myocardial cells may be PDCD4, calcineurin (CaN) and promoter protein-related protein-1 (Drp-1) (Chua et al. 2016; Jia et al. 2016; Wang et al. 2011). SOX6 plays a crucial role in cell and organ development (Kawasaki et al. 2015; Panman et al. 2014). Studies have confirmed that miR-499-5p induced the up-regulation of type-I fiber number by inhibiting SOX6 (van Rooij et al. 2009). Wang et al. confirmed that miR-499-5p and SOX6 expressions were negatively correlated, and that miR-499-5p could regulate porcine myofiber specification by regulating SOX6 expression (Wang et al. 2017). These studies indicated that miR-499-5p exerted its biological function by regulating SOX6. In the present study, we confirmed that SOX6 was a target gene of miR-499-5p, and that miR-499-5p inhibited H/R-induced apoptosis of H9c2 cells by regulating SOX6. Zhu et al. demonstrated that miR-16 could inhibit cell apoptosis by regulating RECK and SOX6 (Zhu et al. 2014). These studies suggested that miRNA could protect cardiomyocytes by down-regulating SOX6 expression. In addition, several studies have found that miRNA promoted the proliferation, migration and invasion of cancer cells and inhibited apoptosis by targeting SOX6 (Li and Wang 2018; Jin et al. 2018).

It has been found that the levels of LDH and MDA were increased in the damaged myocardium (Jiang et al. 2018; Wu et al. 2016). LDH, which exists in almost all tissues, is the most abundant content in the heart and kidney. Parhamifar et al. believed that LDH release was an early event of cell necrosis and a late event of apoptosis (Parhamifar et al. 2013). Researchers also showed that the level of MDA was increased in H/R-induced myocardial injury (Chen et al. 2018). In our investigation, after hypoxia induction, the protein expressions of LDH and MDA were up-regulation, while the LDH and MDA levels were inhibited by up-regulating miR-499-5p, and such inhibitory effects could be reversed by SOX6. These results suggested that miR-499-5p could prevent myocardial H/R injury by reducing cell necrosis through inhibiting SOX6 expression.

To further explore the mechanism of miR-499-5p in H/R-induced H9c2 cells, the levels of related apoptotic proteins were determined by western blot. Bcl-2, Bax and caspase-3 are key regulators of apoptosis, and apoptosis mediated by the dysregulation of such three genes underlies a plethora of diseases (Fong et al. 2017; Mitupatum et al. 2016). Bcl-2 and Bax are important genes in Bcl-2 gene family, and Bcl-2 inhibits apoptosis, while Bax promotes apoptosis. Caspase-3, which is one of the members of the interleukin-1-beta invertase (ICE) family, is a main executive caspase in apoptosis and a common downstream effector of multiple apoptotic pathways (Odonkor and Achilefu 2009). In our study, high expression of miR-499-5p up-regulated Bcl-2 expression and down-regulated Bax and caspase-3 expressions under hypoxia, while SOX6 partially reversed the inhibitory effect of miR-499-5p on cell apoptosis, indicating that miR-499-5p inhibited cell apoptosis by regulating the expression of SOX6. It has been found that the regulation of Bax and Bcl-2 on apoptosis is not only affected by their expressions, but also by Bcl-2/Bax ratio (Wang et al. 2014b). MiR-499-5p may play an anti-apoptosis role by regulating the proportion of Bcl-2 and Bax protein expressions in cells. In addition, the Bcl-2 can form heterodimer with Bax and inhibit the activation of downstream caspase-9 and its effector protein caspase-3 by blocking the release of cytochrome c, thus effectively inhibiting the occurrence of apoptosis (Xu et al. 2016). Jia et al. found that miR-499 protected cardiomyocytes apoptosis induced by LPS via regulating the SOX6/PDCD4-BCL-2 signaling pathway (Jia et al. 2016). One study showed that high expression of miR-142-3p could promote the expression of Bcl-2 and inhibit the expression of caspase-3 (Wang et al. 2016b).

The present study presents some limitations, for example, the morphological change of apoptosis was not investigated. Furthermore, this study was performed in vitro, therefore, further in vivo experiments are required to confirm the present observations.

In conclusion, we demonstrated that miR-499-5p suppressed H/R-induced apoptosis of cardiomyocytes by targeting SOX6 expression, suggesting that miR-499-5p/SOX6 pathway may have a potential therapeutic target for the treatment of AMI.

## References

[CR1] Agiannitopoulos K (2018). Expression of miR-208b and miR-499 in greek patients with acute myocardial infarction. In Vivo.

[CR2] Bowles J, Schepers G, Koopman P (2000). Phylogeny of the SOX family of developmental transcription factors based on sequence and structural indicators. Dev Biol.

[CR3] Chen JQ (2017). MicroRNA expression profiles identify disease-specific alterations in systemic lupus erythematosus and primary Sjogren’s syndrome. PloS ONE.

[CR4] Chen M, Wang X, Hu BO, Zhou J, Wang X, Wei W, Zhou H (2018). Ursolic acid stimulates UCP2 expression and protects H9c2 cells from hypoxia-reoxygenation injury via p38 signaling. J Biosci.

[CR5] Cheng J (2018). MicroRNA-449a inhibition protects H9C2 cells against hypoxia/reoxygenation-induced injury by targeting the notch-1 signaling pathway. Cell Physiol Biochem.

[CR6] Chua SK, Wang BW, Lien LM, Lo HM, Chiu CZ, Shyu KG (2016). Mechanical stretch inhibits MicroRNA499 via p53 to regulate calcineurin-A expression in rat cardiomyocytes. PloS ONE.

[CR7] Du H, Hao J, Liu F, Lu J, Yang X (2015). Apigenin attenuates acute myocardial infarction of rats via the inhibitions of matrix metalloprotease-9 and inflammatory reactions. Int J Clin Exp Med.

[CR8] Feng Y (2016). WDR26 promotes mitophagy of cardiomyocytes induced by hypoxia through Parkin translocation. Acta Biochim Biophys Sin (Shanghai).

[CR9] Fong HY, Abd Malek SN, Yee HS, Karsani SA (2017). Helichrysetin Induces DNA Damage that Triggers JNK-Mediated Apoptosis in Ca Ski Cells. Pharm Mag.

[CR10] Greco S, Gaetano C, Martelli F (2014). HypoxamiR regulation and function in ischemic cardiovascular diseases. Antioxid Redox Signal.

[CR11] Hernandez-Resendiz S, Chinda K, Ong SB, Cabrera-Fuentes H, Zazueta C, Hausenloy DJ (2018). The Role of redox dysregulation in the inflammatory response to acute myocardial ischaemia-reperfusion injury—adding fuel to the fire. Curr Med Chem.

[CR12] Huang Z, Wu S, Kong F, Cai X, Ye B, Shan P, Huang W (2017). MicroRNA-21 protects against cardiac hypoxia/reoxygenation injury by inhibiting excessive autophagy in H9c2 cells via the Akt/mTOR pathway. J Cell Mol Med.

[CR13] Jia Z (2016). SOX6 and PDCD4 enhance cardiomyocyte apoptosis through LPS-induced miR-499 inhibition. Apoptosis.

[CR14] Jiang WB, Zhao W, Chen H, Wu YY, Wang Y, Fu GS, Yang XJ (2018). Baicalin protects H9c2 cardiomyocytes against hypoxia/reoxygenation-induced apoptosis and oxidative stress through activation of mitochondrial aldehyde dehydrogenase 2. Clin Exp Pharmacol Physiol.

[CR15] Jin RH, Yu DJ, Zhong M (2018). MiR-1269a acts as an onco-miRNA in non-small cell lung cancer via down-regulating SOX6. Eur Rev Med Pharm Sci.

[CR16] Kawasaki K (2015). Expression of Sox genes in tooth development. Int J Dev Biol.

[CR18] Li X (2013). MiR-499 regulates cell proliferation and apoptosis during late-stage cardiac differentiation via Sox6 and cyclin D1. PloS One.

[CR43] Li Y, Lu J, Bao X, Wang X, Wu J, Li X, Hong W (2016). MiR-499-5p protects cardiomyocytes against ischaemic injury via anti-apoptosis by targeting PDCD4. Oncotarget.

[CR17] Li Z, Wang Y (2018). miR-96 targets SOX6 and promotes proliferation, migration, and invasion of hepatocellular carcinoma. Biochem Cell Biol.

[CR19] Liang ZG, Yao H, Xie RS, Gong CL, Tian Y (2018). MicroRNA20b5p promotes ventricular remodeling by targeting the TGFbeta/Smad signaling pathway in a rat model of ischemiareperfusion injury. Int J Mol Med.

[CR20] Liu L, Yuan Y, He X, Xia X, Mo X (2017). MicroRNA-1 upregulation promotes myocardiocyte proliferation and suppresses apoptosis during heart development. Mol Med Rep.

[CR21] Matkovich SJ, Hu Y, Eschenbacher WH, Dorn LE, Dorn GW (2012). Direct and indirect involvement of microRNA-499 in clinical and experimental cardiomyopathy. Circ Res.

[CR22] Mitupatum T, Aree K, Kittisenachai S, Roytrakul S, Puthong S, Kangsadalampai S, Rojpibulstit P (2016). mRNA expression of Bax, Bcl-2, p53, cathepsin B, caspase-3 and caspase-9 in the HepG2 cell line following induction by a novel monoclonal Ab Hep88 mAb: cross-talk for paraptosis and apoptosis. Asian Pac J Cancer Prev.

[CR23] Odonkor CA, Achilefu S (2009). Modulation of effector caspase cleavage determines response of breast and lung tumor cell lines to chemotherapy. Cancer Invest.

[CR24] Olivieri F (2013). Diagnostic potential of circulating miR-499-5p in elderly patients with acute non ST-elevation myocardial infarction. Int J Cardiol.

[CR25] Panman L (2014). Sox6 and Otx2 control the specification of substantia nigra and ventral tegmental area dopamine neurons. Cell Rep.

[CR26] Parhamifar L, Andersen H, Moghimi SM (2013). Lactate dehydrogenase assay for assessment of polycation cytotoxicity. Methods Mol Biol.

[CR27] Ren L, Wang Q, Chen Y, Ma Y, Wang D (2018). Involvement of MICRORNA-133a in the protective effect of hydrogen sulfide against ischemia/reperfusion-induced endoplasmic reticulum stress and cardiomyocyte apoptosis. Pharmacology.

[CR28] Shieh JT, Huang Y, Gilmore J, Srivastava D (2011). Elevated miR-499 levels blunt the cardiac stress response. PloS ONE.

[CR29] Sluijter JP, van Mil A, van Vliet P, Metz CH, Liu J, Doevendans PA, Goumans MJ (2010). MicroRNA-1 and -499 regulate differentiation and proliferation in human-derived cardiomyocyte progenitor cells. Arterioscler Thromb Vasc Biol.

[CR30] van Rooij E (2009). A family of microRNAs encoded by myosin genes governs myosin expression and muscle performance. Dev Cell.

[CR31] Wan Q (2018). miR-499-5p Attenuates Mitochondrial Fission and Cell Apoptosis via p21 in Doxorubicin. Cardiotoxic Front Genet.

[CR32] Wang GK (2010). Circulating microRNA: a novel potential biomarker for early diagnosis of acute myocardial infarction in humans. Eur Heart J.

[CR33] Wang JX (2011). miR-499 regulates mitochondrial dynamics by targeting calcineurin and dynamin-related protein-1. Nat Med.

[CR34] Wang J (2014). miR-499 protects cardiomyocytes from H 2O 2-induced apoptosis via its effects on Pdcd4 and Pacs2. RNA Biol.

[CR35] Wang Y, Zhang H, Chai F, Liu X, Berk M (2014). The effects of escitalopram on myocardial apoptosis and the expression of Bax and Bcl-2 during myocardial ischemia/reperfusion in a model of rats with depression. BMC Psychiatry.

[CR36] Wang J, Aung LH, Prabhakar BS, Li P (2016). The mitochondrial ubiquitin ligase plays an anti-apoptotic role in cardiomyocytes by regulating mitochondrial fission. J Cell Mol Med.

[CR37] Wang Y, Ouyang M, Wang Q, Jian Z (2016). MicroRNA-142-3p inhibits hypoxia/reoxygenationinduced apoptosis and fibrosis of cardiomyocytes by targeting high mobility group box 1. Int J Mol Med.

[CR38] Wang XY (2017). MicroRNA-499-5p regulates porcine myofiber specification by controlling Sox6 expression. Animal.

[CR39] Wu D, Jiang H, Chen S, Zhang H (2015). Inhibition of microRNA-101 attenuates hypoxia/reoxygenationinduced apoptosis through induction of autophagy in H9c2 cardiomyocytes. Mol Med Rep.

[CR40] Wu D (2016). In vitro evaluation of aspirin-induced HspB1 against heat stress damage in chicken myocardial cells. Cell Stress Chaperones.

[CR41] Xin Y, Yang C, Han Z (2016). Circulating miR-499 as a potential biomarker for acute myocardial infarction. Ann Transl Med.

[CR42] Xu G, Kuang G, Jiang W, Jiang R, Jiang D (2016). Polydatin promotes apoptosis through upregulation the ratio of Bax/Bcl-2 and inhibits proliferation by attenuating the beta-catenin signaling in human osteosarcoma cells. Am J Transl Res.

[CR44] Zhang B (2014). MicroRNA-92a inhibition attenuates hypoxia/reoxygenation-induced myocardiocyte apoptosis by targeting Smad7. PloS ONE.

[CR45] Zhang L (2015). Circulating miR-499 are novel and sensitive biomarker of acute myocardial infarction. J Thorac Dis.

[CR46] Zhang Z, Li H, Chen S, Li Y, Cui Z, Ma J (2017). Knockdown of MicroRNA-122 protects H9c2 cardiomyocytes from hypoxia-induced apoptosis and promotes autophagy. Med Sci Monit.

[CR47] Zhu Y, Xia Y, Niu H, Chen Y (2014). MiR-16 induced the suppression of cell apoptosis while promote proliferation in esophageal squamous cell carcinoma. Cell Physiol Biochem.

[CR48] Zhu J (2016). Ischemic postconditioning-regulated miR-499 protects the rat heart against ischemia/reperfusion injury by inhibiting apoptosis through PDCD4. Cell Physiol Biochem.

